# Antegrade Facilitated Antegrade Dissection and Re-Entry: A Novel Solution for Wire Across Nothing Would Follow

**DOI:** 10.1016/j.jaccas.2025.104404

**Published:** 2025-07-30

**Authors:** Saroj Neupane, Simon Walsh

**Affiliations:** Division of Cardiology, Banner University Medical Center, Phoenix, Arizona, USA

**Keywords:** antegrade dissection and re-entry, balloon uncrossable, chronic total occlusion, percutaneous coronary intervention

## Abstract

A common challenge in percutaneous coronary interventions of chronic total occlusions (CTO) is balloon uncrossable lesions. It occurs when there is failure in crossing the lesion with a balloon or microcatheter after successfully crossing with a guidewire. This challenge is also not uncommon in non-CTO lesions with heavy calcification and tortuosity. Several techniques have been described to overcome this challenge during percutaneous coronary interventions of CTO and non-CTO lesions. We describe 2 cases illustrating the novel use of a Stingray catheter to perform antegrade dissection and re-entry in balloon uncrossable lesions.

## Graphical Abstract

**Figure:**
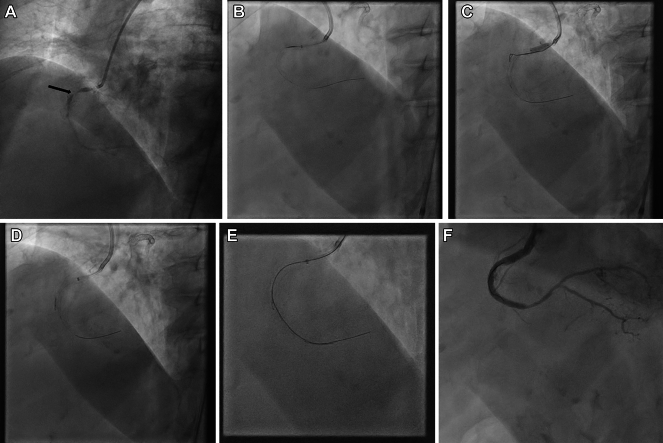

A common challenge in percutaneous coronary interventions (PCI) of chronic total occlusions (CTO) is inability to cross the lesion *with a balloon or microcatheter after successfully crossing with a guidewire*.^1^ This is commonly referred as balloon uncrossable (BU) lesions. This is also not uncommon in non-CTO lesions especially with heavy calcification and tortuosity. Several techniques have been described to overcome this challenge during PCI of both CTO and non-CTO lesions.^2^ The following cases illustrate the novel use of a Stingray low profile catheter (Boston Scientific) to perform facilitated antegrade dissection and re-entry (ADR) in BU lesions.

**Take-Home Message**

• Facilitated antegrade dissection and re-entry can be performed safely in balloon uncrossable lesions when routine techniques fail.

## Case 1

**Figure 1:**
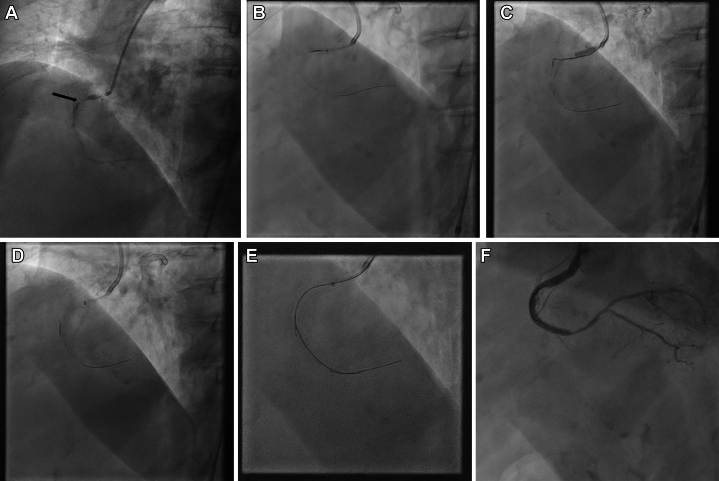
Coronary Angiogram and Intervention (A) Right coronary artery, subtotal occlusion in proximal segment (arrow) with poor distal filling and heavy calcification. (B) Crossing of the lesion with a medium tip load polymer jacketed wire supported by a microcatheter. (C) Balloon-assisted subintimal entry proximal to the lesion to gain access to the subintimal space with a 1:1 size noncompliant balloon. (D) Stingray low profile catheter over the subintimal wire into the distal landing zone. (E) Facilitated antegrade dissection and re-entry with the stick and drive technique using the true lumen wire as a marker. (F) Final result after intravascular ultrasound–guided stenting.

An 81-year-old woman with severe multivessel coronary artery disease who recently had a PCI of the left anterior descending and left circumflex arteries was still experiencing ongoing symptoms. Therefore, the patient was brought back for a PCI of the right coronary artery. The right coronary artery had a subtotal occlusion with heavy calcification in the proximal segment (Figure 1A, Video 1A). The lesion was crossed with a medium tip load polymer jacketed wire supported by a microcatheter (Figure 1B, Video 1B). We were unable to advance the microcatheter across the lesion despite attempting conventional techniques such as balloon assisted microdissection etc. Balloon-assisted subintimal entry was performed proximal to the lesion to gain access to the subintimal space (Figure 1C, Video 1C). External cap crush was then attempted without success. A Gladius Mongo wire (Asahi Intecc) in subintimal space was knuckled distal to the lesion, leaving the true lumen wire in place. A Stingray low profile catheter was advanced over the subintimal wire to the distal landing zone (Figure 1D). Facilitated ADR was performed with the stick and drive technique (Figure 1E, Video 1D), using the true lumen wire as a marker. The intravascular ultrasound guided PCI was then performed in a standard fashion (Figure 1F, Video 1E).

## Case 2

**Figure 2:**
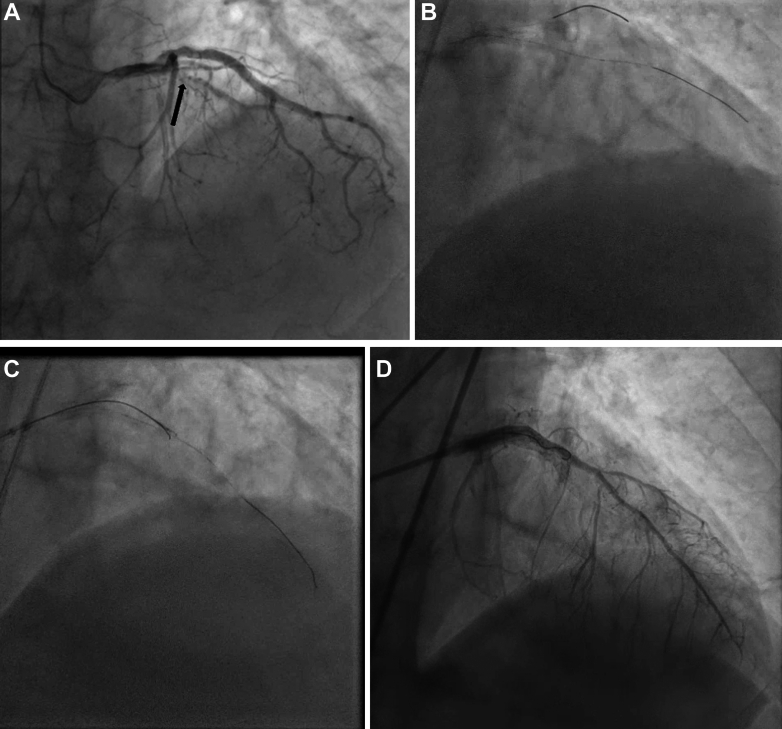
Coronary Angiogram and Intervention (A) Left anterior descending (LAD), subtotally occluded with heavy calcification (arrow) with poor distal filling. (B) Crossing of the lesion with a medium tip load polymer jacketed wire supported by a microcatheter. (C) External cap crush with a 1:1 size noncompliant balloon over a Gladius Mongo wire in the subintimal space. (D) Facilitated antegrade dissection and re-entry with the stick and drive technique using the true lumen wire as a marker. (E) Final result of LAD after stenting.

A 67-year-old man underwent a coronary angiogram for Canadian Cardiovascular Society III angina despite already being on medical therapy. The patient's left anterior descending artery was subtotally occluded with heavy calcification (Figure 2A, Video 2A). The lesion was first crossed with a medium tip load polymer jacketed wire supported by a microcatheter (Figure 2B, Video 2B). We were unable to advance the microcatheter across the lesion. The algorithmic solution for BU lesions, including balloon-assisted microdissection and laser atherectomy, was followed but with no success.^2^ A Gladius Mongo wire was then placed in the subintimal space, and external cap crush with a 1:1 size noncompliant balloon was attempted with no success in crossing the lesion (Figure 2C). The subintimal wire was then knuckled distal to the lesion. A Stingray catheter was advanced over the subintimal wire and placed in the distal the landing zone. Facilitated ADR with a stick and drive technique was performed (Figure 2D, Video 2C) using the true lumen wire as a marker. After successful crossing, intravascular ultrasound–guided PCI was performed in standard fashion (Figure 2E, Video 2D).

## Discussion

Our case series describes the novel application of stingray-based re-entry to solve BU lesions. Several techniques have been described in the past, which include the algorithmic approach by Riley et al^2^ The last resort for this challenge is attempting a retrograde crossing. However, the success of retrograde crossing depends on the presence of intervention collaterals, which may not present in non-CTO lesions.^3^ The retrograde approach also has a higher complication rate than antegrade techniques.^3^ This technique provides an effective and safer alternative to the retrograde approach, especially if the distal target vessel is of good quality. One challenge with the technique is the development of a subintimal hematoma, which can cause ischemia by compressing true lumen especially in non-CTO lesions. It also reduces the chance of successful re-entry. Therefore, it is critical to take measures to reduce subintimal hematoma, such as plugging inflow with a guide extension catheter, minimizing exchanges, and subintimal transcatheter withdrawal. We successfully used a guide extension catheter to minimize subintimal hematoma in case 1. We were unable to use guide extension for this purpose in case 2 because of severe ischemia as evidenced by chest pain, electrocardiogram changes, and hypotension. Therefore, we had to perform subintimal transcatheter withdrawal for a prolonged period to facilitate re-entry. The other advantage of this technique is that the true lumen wire can serve as a marker and show vessel course. As a result, the re-entry can be performed with higher confidence even in the absence of collateral filling or without contralateral or ipsilateral injection. It can be of value in non-CTO lesions, like the cases discussed above, as well as in CTO lesions where contralateral filling of the distal vessel is not robust.

## Funding Support and Author Disclosures

Drs Neupane and Walsh serve as consultants for Boston Scientific Corporation and Asahi Intecc USA, Inc.

## Appendix

Video 1 XXX (A) Right coronary artery, subtotal occlusion in proximal segment (arrow) with poor distal filling and heavy calcification. (B) Crossing of the lesion with a medium tip load polymer jacketed wire supported by a microcatheter. (C) Balloon-assisted subintimal entry proximal to the lesion to gain access to the subintimal space with a 1:1 size noncompliant balloon. (D) Facilitated ADR with the stick and drive technique using the true lumen wire as a marker. (E) Final result after intravascular ultrasound–guided stenting.

Video 2 XXX (A) Left anterior descending (LAD), subtotally occluded with heavy calcification with poor distal filling. (B) Crossing of the lesion with a medium tip load polymer jacketed wire supported by a microcatheter. (C) Facilitated ADR with the stick and drive technique using the true lumen wire as a marker. (D) Final result of LAD after stenting.
